# Overcoming blood brain barrier with a dual purpose Temozolomide loaded Lactoferrin nanoparticles for combating glioma (SERP-17-12433)

**DOI:** 10.1038/s41598-017-06888-4

**Published:** 2017-07-26

**Authors:** Sonali Kumari, Saad M. Ahsan, Jerald M. Kumar, Anand K. Kondapi, Nalam M. Rao

**Affiliations:** 1 Department of Biotechnology and Bioinformatics, School of Life Sciences, University of Hyderabad, Prof. C. R. Rao Road, Gachibowli, Hyderabad, 500 046 Telangana State India; 2 Centre for Cellular and Molecular Biology (CCMB), Council of Scientific and Industrial Research, Uppal Road, Hyderabad, 500 007 Telangana State India

## Abstract

Targeted delivery of drugs to the brain is challenging due to the restricted permeability across the blood brain barrier (BBB). Gliomas are devastating cancers and their positive treatment outcome using Temozolomide (TMZ) is limited due to its short plasma half-life, systemic toxicity and limited access through the blood-brain barrier (BBB). Nanoparticles made of Lactoferrin (Lf) protein, have been shown to enhance the pharmacological properties of drugs. Here, we report the specific ability of Lf nanoparticles to cross BBB and target over-expressed Lf receptors on glioma for enhanced TMZ delivery. TMZ-loaded Lf nanoparticles (TMZ-LfNPs) were prepared by our previously reported sol-oil method. While the Lf protein in the NP matrix aids in transcytosis across the BBB and preferential tumor cell uptake, the pH responsiveness leads to TMZ release exclusively in the tumor microenvironment. Delivery through LfNPs results in an enhanced and sustained intracellular concentration of TMZ in GL261 cells *in vitro* along with improving its *in vivo* pharmacokinetics and brain accumulation. TMZ-LfNPs treatment results in a significant reduction of tumor volume, higher tumor cell apoptosis and improved median survival in glioma bearing mice. These results demonstrate that LfNPs present an efficient TMZ delivery platform for an effective treatment of gliomas.

## Introduction

Positive clinical outcome of glioma, the most common malignant brain tumor, is dismally poor^1^. This is mainly due to poor prognosis and lack of effective therapeutic options after diagnosis. Originating from astrocytes, a type of glial cells in brain, glioma infiltrates into other brain tissues as well^2^. The exact cause(s) of glioma are unknown and several genes have been implicated in its origin^3^. The major reasons behind treatment failures are the proliferative nature of the tumor, the inaccessibility of brain to a number of small and large molecular drugs and high chances of recurrence after treatment^4^.

Temozolomide, a DNA alkylating agent, is the preferred drug for glioma treatment and has been shown to provide clinically meaningful survival benefits in patients^5^. However, a prolonged therapy leads to TMZ resistance and poor responsiveness to subsequent treatments. This is mainly due to an induction of enhanced levels of DNA damage repair enzymes^6^, thus leading to tumor recurrence in 60–75% of patients^5, 7^. In addition, TMZ requires continuous administration due to its low solubility in physiological media and shorter plasma half-life (1.8 h)^8^. While higher doses of TMZ may result in positive outcomes by way of causing extensive tumor kill, the resulting toxic effects of TMZ does not permit dose escalation and results in haematological toxicity^9^, acute cardiomyopathy^10^, oral ulceration, hepatotoxicity^11^ and pneumocystis pneumonia^12^ and ultimately resulting in discontinuation of therapy. The limitations associated with TMZ therapy highlights the need for a delivery vehicle to increase the therapeutic index of TMZ. Several carriers of TMZ, including liposomes, solid lipid nanoparticles, polymers etc. have been tested for their efficacy. The success of these formulations was however been limited due to lack of tumor specific delivery of TMZ^11, 13^. A drug delivery system for glioma therapy should be targetable to the tumor with an ability to cross the BBB.

Lactoferrin (Lf) is an 80 kDa cationic protein belonging to the transferrin family. It is an iron carrying protein with multiple physiological roles including host-defence against infection and inflammation^14, 15^. Receptors of Lf are known to be over-expressed in glioma cells and on brain endothelial cells^16–19^. Lf is known for its ability to cross BBB and this property has been extensively used for targeted delivery of several drugs to the brain^19, 20^. Our group has previously reported the benefits of delivering several drugs using Lf nanoparticles^21–23^. These Lf nanoparticles were prepared with Lf as a sole matrix, by sol-oil method, without the involvement of any chemical modifications of Lf.

We hypothesize that nanoparticles prepared exclusively with Lf as sole matrix with entrapped TMZ would retain its ligand properties, efficiently cross the BBB and deliver the drug in a targeted fashion to the glioma. Further, due to the entrapment of TMZ in NPs, the toxic side effects of the drug may be reduced. The current study aims to develop TMZ loaded lactoferrin nanoparticles (TMZ-LfNPs) and their characterization. Using a combination of *in vitro* and *in vivo* methods we have evaluated the BBB crossing, glioma targeting and enhanced therapeutic efficacy of TMZ when delivered through LfNPs.

## Results

### Preparation and characterization of TMZ-LfNPs

**Figure 1:**
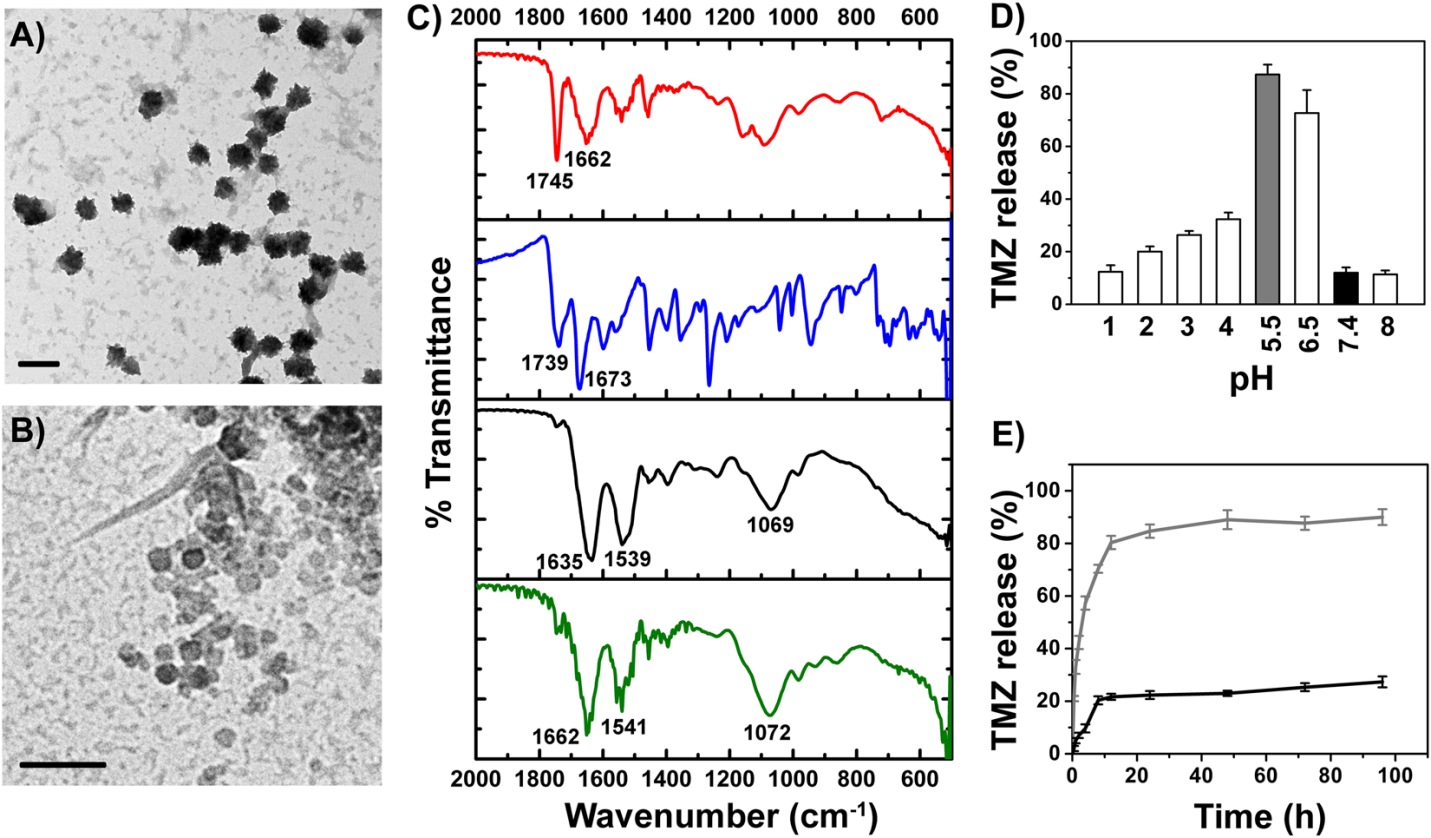
TEM analysis of particles incubated at **(A)** pH 7.4 and **(B)** pH 5.5 (scale bar 200 nm). **(C)** FT-IR spectra of free Lf (black), LfNPs (green), TMZ (blue) and TMZ-LfNPs (red). **(D)** Release of TMZ from LfNPs studied at different pH values. pH 5.5 and pH 7.4 are highlighted in grey and black respectively. **(E)** Cumulative TMZ release from LfNPs at pH 5.5 (grey) and pH 7.4 (black). Each data points were repeated in triplicates (n = 3) and presented as Mean ± Standard Deviation (S.D).

TMZ-LfNPs were prepared by a previously established sol-oil method^24, 25^. The TMZ-LfNPs, as characterized by TEM and DLS, showed a spherical morphology and uniform size distribution in the range of 70 ± 10 nm (TEM) **(**Fig. 1A **)**. The hydrodynamic diameter, as studied by DLS, was found to be 160 ± 13.3 nm. The larger size obtained by DLS could be attributed to the contribution of the associated hydration shell of the nanoparticles. The polydispersity index (PDI) and zeta-potential of the particles were 0.24 ± 0.1 and −2.5 ± 0.2 mV respectively, suggesting a narrow size distribution and slight negative (near neutral) surface charge of the particles. Fluorescein loaded LfNPs (FL-LfNPs) were used for confocal experiments and their physico-chemical properties (size and zeta-potential) **(**Supplementary Fig. 1A and B) were found to be similar to the TMZ-LfNPs. The size of the nanoparticles prepared in several batches and also at different starting protein concentrations (10–50 mg/ml) was found to be very reproducible (Size 170.3 ± 12.8 nm, n = 6). The stability of the LfNPs was assessed by monitoring the hydrodynamic diameter using DLS. The nanoparticles, were found to be stable when stored in PBS and in the presence of serum (10%) at 4 °C, for a period of 2 months **(**Supplementary Fig. 2 **)**.

### Assessment of encapsulation efficiency

The encapsulation efficiency (EE) of TMZ in LfNPs was found to be 42 ± 4.9% (n = 3) with a drug loading content (DLC) of 17.5 ± 2.5% (n = 3). The high EE and DLC achieved in the present study, as compared to the previous reports^13, 26, 27^ was probably due to the partitioning of TMZ in the hydrophobic patches of Lf in the LfNPs.

### FT-IR spectral analysis

The entrapment of TMZ in the LfNPs was confirmed by the FT-IR spectral analysis **(**Fig. 1C **)**. FT-IR spectrum of TMZ-LfNPs showed characteristic peaks of Lf and TMZ **(**Supplementary Table 1 **)** suggesting that the drug is entrapped in the particle without any interactions with Lf protein - a feature important to preserve TMZ efficacy and the LfR targeting ability of Lf.

### pH dependent release of TMZ from LfNPs

The release of the encapsulated TMZ, speculated to be through diffusion, was found to be enhanced under mildly acidic pH conditions. TMZ release (60–70%) from LfNPs was found to be significant in buffers with a slightly acidic pH (maximum at pH 5.5) **(**Fig. 1D, **grey bar)**. However, the release of TMZ from LfNPs at physiological pH (7.4), was found to be less (10–20%) **(**Fig. 1D, **black bar)**. The release of TMZ at acidic pH could be attributed to the shrinkage of LfNPs under these conditions. The hydrodynamic diameter of LfNPs was found to be reduced to 80 ± 10.6 nm at pH 5.5 as compared to160 ± 13.3 nm obtained at pH 7.4. TEM analysis also revealed a reduced size of 40 ± 15 nm **(**Fig. 1B **)** at pH 5.5 as compared to 70 ± 10 nm observed at pH 7.4 **(**Fig. 1A **)**. However, no significant changes were observed in the zeta-potential of the LfNPs at these two pH values.

The TMZ release kinetics from LfNPs was studied over a period of 72 h. As shown in Fig. 1E **(gray curve)**, TMZ release from LfNPs at pH 5.5 exhibited a biphasic profile with an initial rapid release phase (about 60–70%) in the first 10 h, followed by a slow and sustained release till 72 h (15 to 20%). However, the release of TMZ at pH 7.4 was found to be minimal and remained low over a period of 72 h **(**Fig. 1E, **black curve)**.

### Receptor-mediated endocytosis of LfNPs

**Figure 2:**
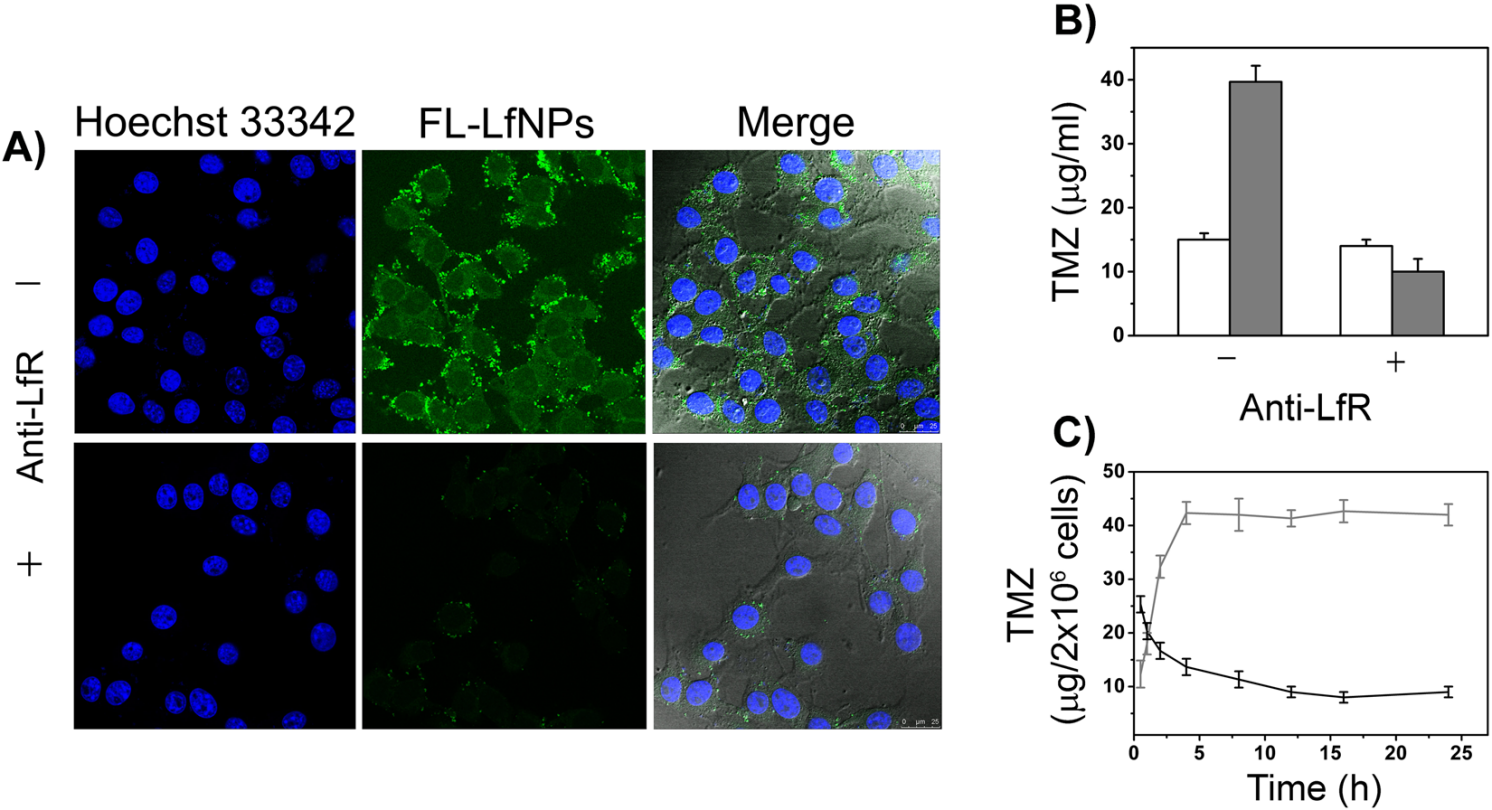
(**A**) FL-LfNPs uptake in GL261 cells in the absence (top panel) and presence (lower panel) of anti-LfR antibodies. (Scale bar = 25 µm). **(B)** Intracellular TMZ concentration, obtained by treating GL261 cells with either free TMZ (white bars) or TMZ-LfNPs (gray bars) in the presence and absence of anti-LfR antibodies. (**C**) Comparative TMZ uptake and clearance by GL261 cells treated with either free TMZ (black curve) or TMZ-LfNPs (grey curve). Each data points were repeated in triplicates (n = 3) and presented as Mean ± Standard Deviation (S.D).

LfRs are known to be over-expressed in the lumen of endothelial cells and surface of glioma cells^16–18^. The expression of LfRs in GL261 cells was studied and compared to liver hepatocellular carcinoma (HepG2) cells, which are reported to be positive for LfR expression^28^. The lung carcinoma cell line (A549), was used as a negative control^29^. As shown in Supplementary Fig. 3, the expression of LfRs in GL261 cells was found to be comparable to that of HepG2 suggesting a significantly high expression of LfR in these cells. The specific role of LfRs in the uptake of LfNPs was thereafter validated by a receptor blocking assay, using anti-LfR antibody. In the presence of anti-LfR antibody, the uptake of LfNPs was found to be significantly decreased **(**Fig. 2A **)**, suggesting a possible role of LfRs in the nanoparticle uptake. The receptor mediated uptake of LfNPs was further validated by estimating the intracellular TMZ levels in GL261cells after receptor blocking by anti-LfR antibody. As shown in Fig. 2B **(grey bars)**, LfR blocking led to a significant decrease in the intracellular TMZ concentrations, when delivered through LfNPs. However, the uptake of free TMZ was found to be unaltered in the presence of anti-LfR antibody **(**Fig. 2B, **white bars)**.

The intracellular TMZ accumulation and clearance rate were studied after treating the GL261 cells with TMZ-LfNPs and compared with free TMZ. Treatment with free TMZ resulted in a sudden increase in the intracellular drug concentrations (up to1 h), followed by a steady decrease up to 5 h **(**Fig. 2C **)**. In contrast, the intracellular levels of TMZ, when delivered through LfNPs, were found to increase gradually up to 4 h and thereafter remained constant up to 24 h. The intracellular TMZ concentrations achieved in TMZ-LfNP treated cells were found to be significantly higher (3-folds) compared to cells incubated with free TMZ. The high and sustained TMZ intracellular concentrations achieved in case of TMZ-LfNPs could be due to the large carrying capacity of LfNPs and a steady release of TMZ from the LfNPs.

### *In vitro* cytotoxicity of TMZ-LfNPs

**Figure 3:**
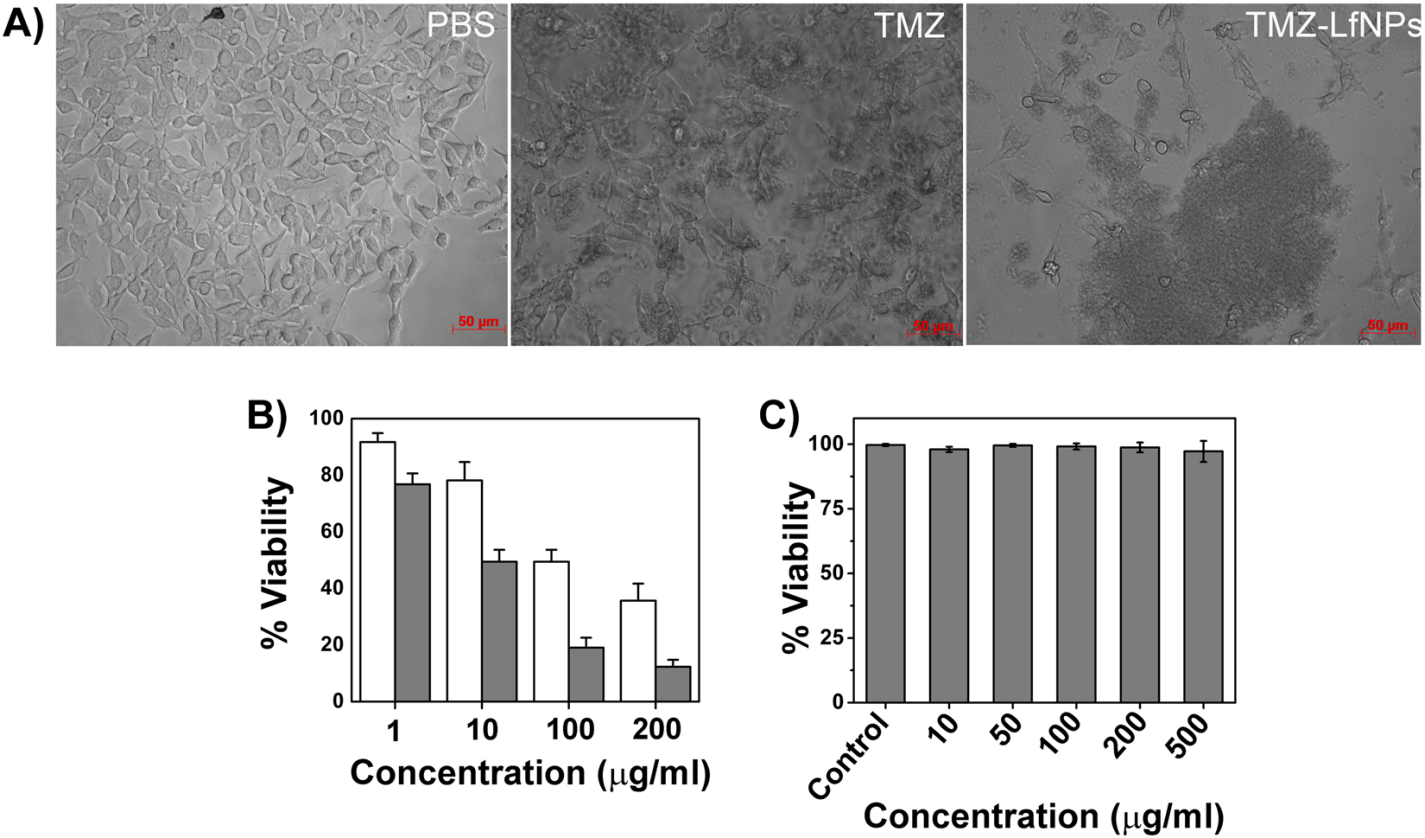
(**A**) Representative microscopic images of untreated cells (left), cells treated with 100 µg ml^−1^ of TMZ (middle) and TMZ-LfNPs-treated cells (equivalent dose) (right). **(B)** GL261 cells treated with different concentrations of TMZ (white bars) and equivalent doses of the TMZ-LfNPs (grey). **(C)** Assessment of biocompatibility of LfNPs. Each data points were repeated in triplicates (n = 3) and presented as Mean ± Standard Deviation (S.D).

To compare the cytotoxic effect of free TMZ and TMZ-LfNPs, a microscopic examination of GL261cells was carried out. Cells treated with TMZ-LfNPs displayed characteristic morphological features of apoptosis and a significant detachment of cells from the surface **(**Fig. 3A, **right panel)**. As compared to free TMZ **(**Fig. 3A, **middle panel)**, treatment with TMZ-LfNPs led to a significant reduction in cell number. The enhanced cytotoxic effect of TMZ-LfNPs was further confirmed by MTT assay **(**Fig. 3B **)**. The IC_50_ values of TMZ **(**Fig. 3B, **white bars)** and TMZ-LfNPs **(**Fig. 3B, **grey bars)** in GL261 cells were found to be 94.3 ± 2.3 and 9.3 ± 1.3 μg ml^−1^ respectively. A 10-fold lower IC_50_ value observed for TMZ-LfNPs, as compared to free TMZ, could be attributed to the enhanced intracellular retention of TMZ when delivered through LfNPs. Further, the vehicle-associated toxicity, i.e., LfNPs, was found to be minimal **(**Fig. 3C **)** even at the highest tested concentration (0.5 mg ml^−1^). LfNPs were also tested (0.01–0.5 mg/ml) on healthy cell line, HEK293 (Human embryonic kidney cells) and were found to be very safe (data not reported).

### Transcytosis of LfNPs across BBB in healthy mice

**Figure 4:**
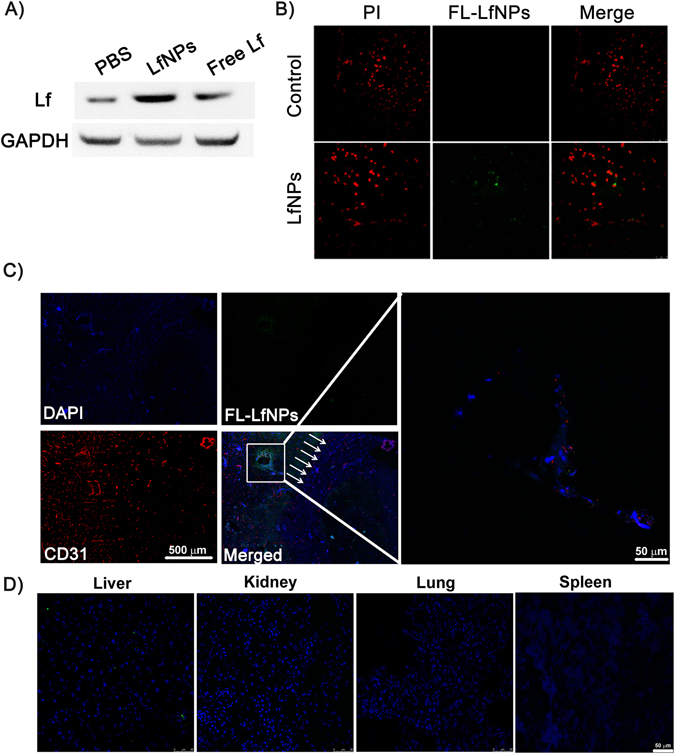
(**A**) Western blot for determining the Lf levels in brain of healthy mice injected with 100 µl of 2 mg ml^−1^ of free Lf and LfNPs (equivalent dose). **(B)** Immunohistochemistry images of brain tissues of control and LfNP-treated animals probed with anti-Lf antibody followed by Alexa Fluor-488 (green) tagged secondary antibody and counter-stained with PI (red). **(C)** Partial projection of a representative tile scan of the mouse brain showing strong fluorescence corresponding to FL-LfNPs (green) in the brain cortex (inset) above the hippocampus (white arrows). Area highlighted in white box is enlarged where fluorescence of FL-LfNPs (green) and brain micro-vessels (stained with anti-CD31 followed by secondary antibody tagged with Cy3 (red)) was visualized. (Scale bar = 500 µm and 50 µm (inset)). **(D)** Organ bio-distribution of FL-LfNPs. Representative confocal images depicts fluorescence signals present in the liver and absent in lung, kidney and spleen.

To assess the transcytosis of LfNPs through BBB, healthy C57BL/6 mice were injected with equivalent amounts of free Lf and LfNPs *via* tail vein injection. Western blot analysis of the brain tissue lysate, obtained at 24 h post-injection, showed significantly higher band intensity in mice injected with LfNPs, as compared to mice injected with free Lf **(**Fig. 4A **)**. The higher brain accumulation of LfNPs could be attributed to the decreased plasma clearance of LfNPs and its ability to cross the BBB. To obtain a cytological evidence for the presence of LfNPs in the brain, animals injected with LfNPs and PBS (negative control) were processed for immunohistochemistry. Confocal images of brain sections showed fluorescence signals (Alexa Fluor-488) corresponding to LfNPs in the brain cortex of mice injected with LfNPs. However, PBS controls did not show any signals (Fig. 4B **)**. To further validate the ability of LfNPs to cross BBB, a tile scan on these brain sections was performed to substantiate the localization of FL-LfNPs along with CD31. As shown in Fig. 4C, distinctly visible FL-LfNPs in the cerebral cortex above the hippocampus region were observed (Fig. 4C, inset in the merged image was zoomed for clarity), indicating transcytosis of LfNPs across the BBB. A lack of fluorescence in the lung, kidney and spleen (Fig. 4D) indicated negligible accumulation of LfNPs in these tissues. However, some fluorescence signals were observed in the liver tissue sections suggesting an accumulation of some fraction of the injected LfNPs in the liver. This could be due to an expression of Lf receptor in the liver tissue^22^. Moreover, liver being a first-pass organ for foreign particles, it is expected to retain some amount of LfNPs^30^.

### Pharmacokinetic studies of TMZ-LfNPs

**Figure 5:**
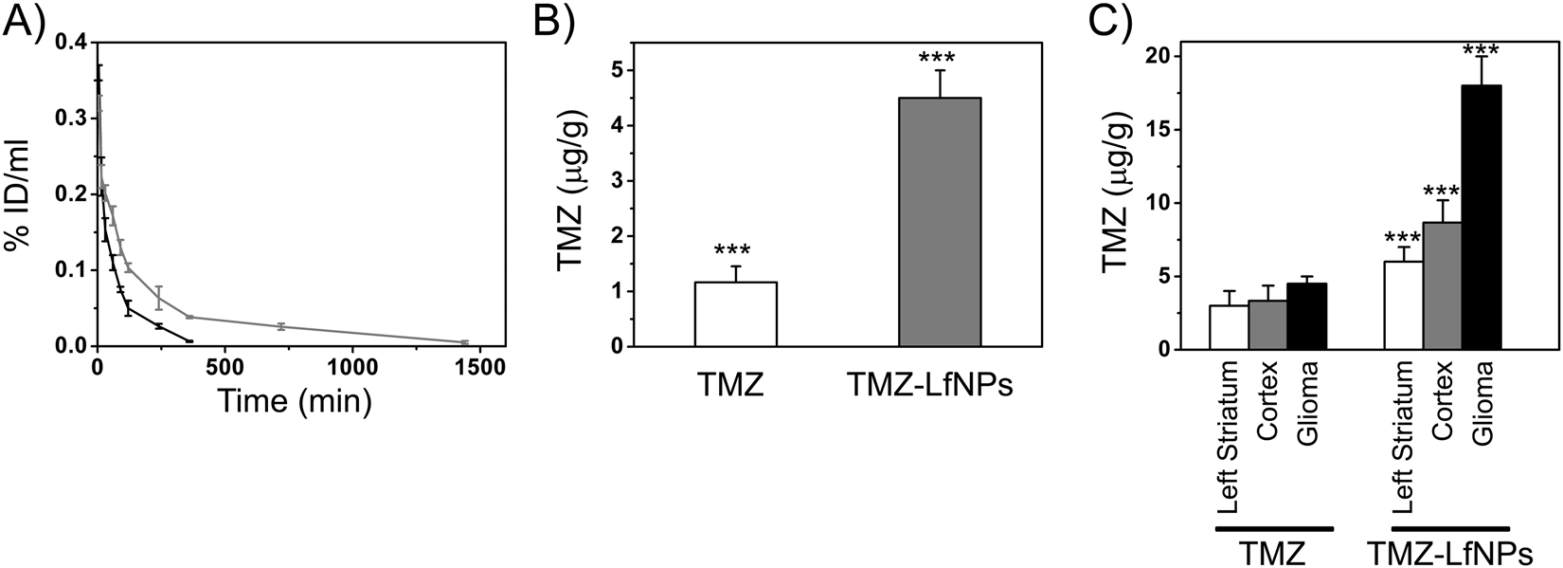
(**A**) Pharmacokinetic studies. % ID/mL of plasma TMZ is plotted versus time after i.v. injection of free TMZ (black) and TMZ-LfNPs (grey) with an equivalent dose of 10 mg kg^−1^ of TMZ in C57BL/6 mice. (**B**) Brain uptake of TMZ in healthy mice 24 h post i.v. injection of TMZ and TMZ-LfNPs with an equivalent dose of 10 mg kg^−1^. Sample data were recorded as Mean ± SD, n = 3. ***P < 0.001. **(C)** Brain uptake of TMZ in glioma bearing mice after i.v. injection of TMZ and TMZ-LfNPs (equivalent dose of 5 mg kg^−1^). ***P < 0.001.

Pharmacokinetic studies of TMZ-LfNPs were carried out by administering a single dose (equivalent TMZ dose of 10 mg kg^−1^ body weight) and compared with free TMZ, into healthy C57BL/6 mice intravenously (IV bolus). The plasma clearance of free TMZ and TMZ-LfNPs followed a biexponential kinetics (Fig. 5A). Free TMZ was cleared rapidly from the circulation with a plasma concentration of approximately 0.21% ID ml^−1^ at 15 min. On the contrary, the plasma TMZ concentration in TMZ-LfNP-administered mice remained high for a longer period. Even 24 h post administration, the plasma concentration for TMZ-LfNPs was found to be 0.02% ID ml^−1^. The pharmacokinetic parameters are listed in Table 1. The TMZ concentrations achieved in the brain (after 24 h), as studied by HPLC **(**Fig. 5B **)**, suggested that delivering TMZ through LfNPs **(**Fig. 5B, **grey bar)** resulted in a significantly higher drug concentration compared to free TMZ (3-fold). Our data suggests that delivery through LfNPs results in an improved pharmacokinetic (PK) profile of TMZ.

**Table 1:** Comparative plasma pharmacokinetic parameters after i.v. injection of free TMZ and TMZ-LfNPs with an equivalent TMZ dose of 10 mg kg^−1^ in mice (n = 3).

Parameters	TMZ	TMZ-LfNPs
T_1/2_ (min)	68 ± 2.13	360 ± 70.86
MRT (min)	88 ± 7.4	367 ± 18.33
AUC (min)*(µg ml^−1^)	4639.66 ± 109.24	10998 ± 754

T_1/2_ - elimination half-life of drug, MRT- mean residence time of drug in the plasma, AUC-The area under curve,

### TMZ-LfNPs brain distribution in glioma mice model

The accumulation of TMZ in the brain tumors was studied in glioma bearing mice. As shown in Fig. 5C, TMZ uptake was found to be highest in the glioma region (right striatum) (Fig. 5C, **black bar)** as compared to other brain areas (left striatum and cortex) **(**Fig. 5C, **white and grey bar)** in TMZ-LfNP-treated mice. The high TMZ tumor concentrations achieved through LfNPs could be attributed to a combined effect of enhanced transcytosis of LfNPs across the BBB, a passive accumulation due to EPR effect and tumor tissue targeting due to the over-expressed LfRs on tumor cells. In contrast, the brain distribution achieved in free TMZ administered mice was found to be significantly lower (p < 0.001) and uniform across different brain regions.

### Tumor regression studies in glioma mice

**Figure 6:**
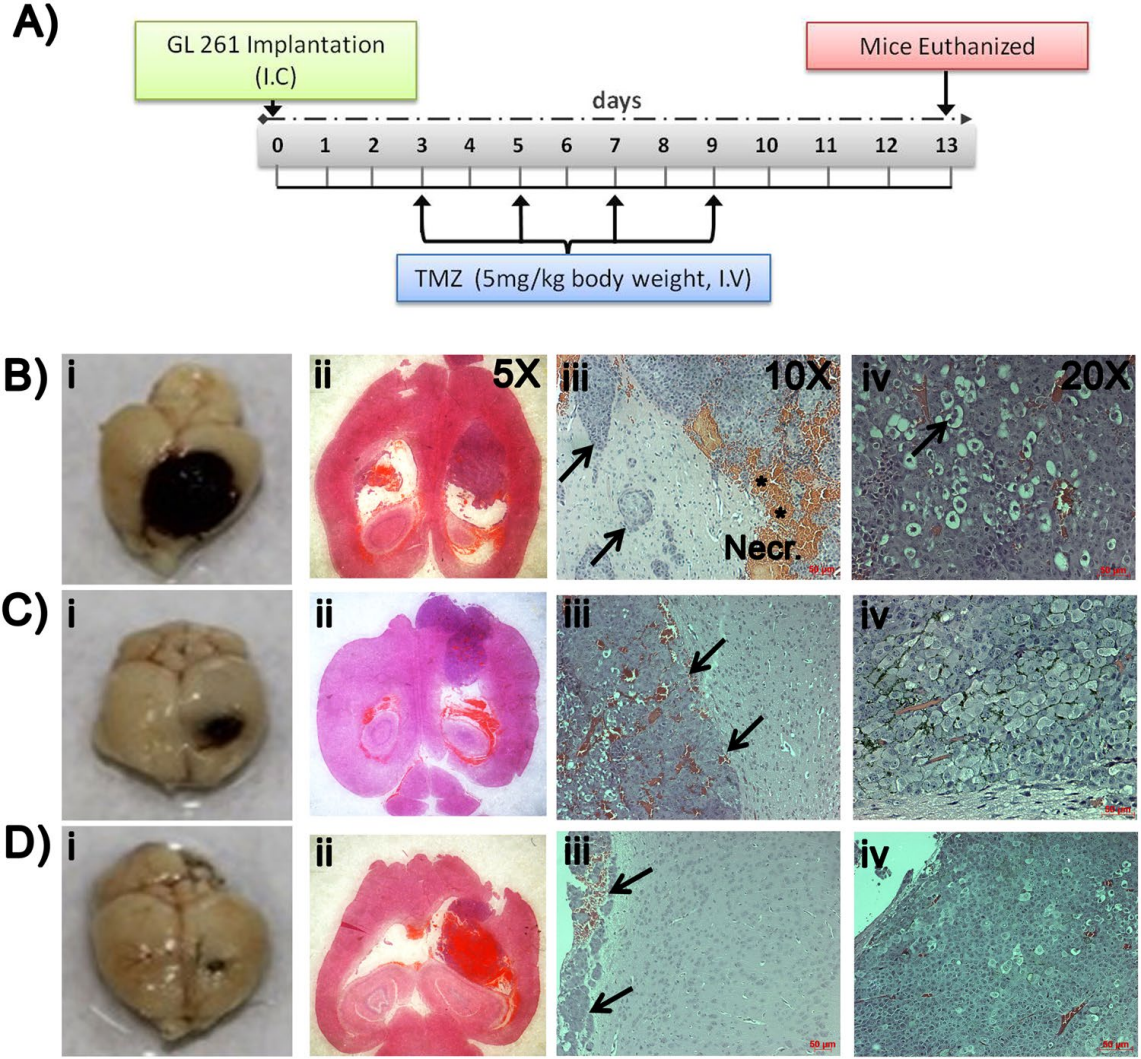
(**A**) Treatment schedule. Photomicrographs of brain (i) and histological brain sections at 5X (ii), 10X (iii) and 20X (iv) magnification obtained from brain dissected from glioma bearing mice treated with PBS **(B)**, TMZ **(C)**, and TMZ-LfNPs **(D)**. (Necr. represents necrotic area with numerous penetrating tumor cells and blood vessels in the periphery of the tumor cell islets (arrows) as well as areas of haemorrhage (asterisks).

To compare the anti-tumor effect of TMZ-LfNPs with free TMZ, glioma bearing mice were randomly divided into three treatment groups (n = 3 mice) as described in the method section and in the treatment schedule (Fig. 6A **)**. On the 14^th^ day when all the PBS treated mice succumbed to death, mice in other groups were also sacrificed and the brain tissues were removed and processed for histology and molecular analysis.

As evident from Fig. 6B, **(i-iv)**, PBS treated mice showed a huge tumor burden, infiltrating tumor cells surrounded by large necrotic areas and numerous atypical cells. Glioma bearing mice treated with free TMZ showed reduced infiltration of tumor and caused moderate shrinkage of the tumor relative to PBS treated mice **(**Fig. 6C, **(i-iv)**. However, the tumor burden was found to be negligible and glioma cells were replaced by normal brain parenchyma cells in mice treated with TMZ-LfNPs **(**Fig. 6D, **(i-iv))**.

**Figure 7:**
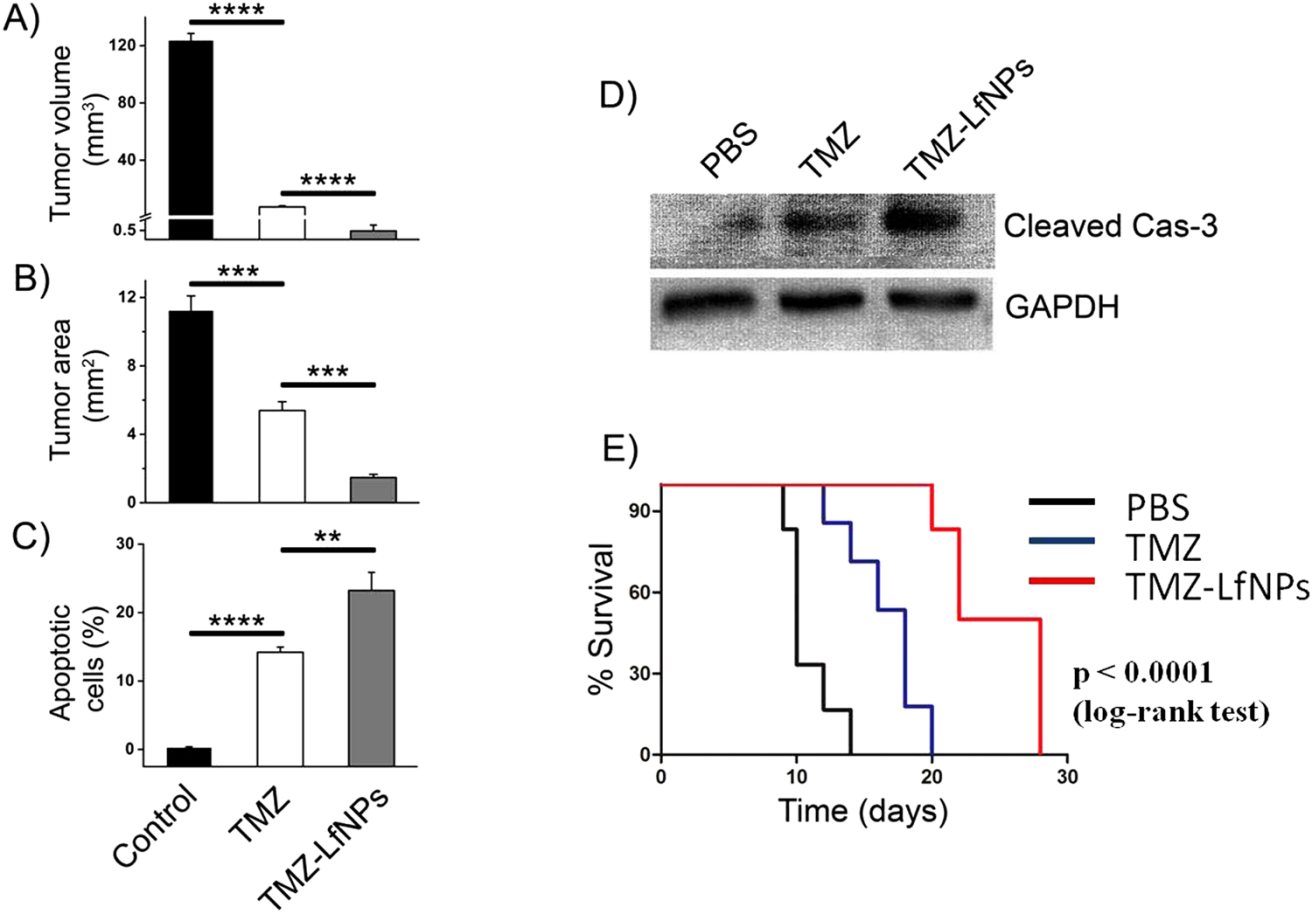
Tumor volume **(A)** and tumor area **(B)** in untreated mice (black bar) and mice treated with TMZ (white bar) and TMZ-LfNPs (grey bar). **(C)** Quantification of apoptosis in untreated mice (black bar) and mice treated with TMZ (white bar) and TMZ-LfNPs (gray bar). (n = 3, **p < 0.01, ***p < 0.001, ****p < 0.0001. **(D)** Western blot of cleaved Caspase-3 in representative tumor lysates. GAPDH is used as a loading control. Full length blot has been given in Supplementary Information **(E)** Kaplan-Meier survival curve; comparison of survival in glioma bearing mice treated with: PBS (black curve), TMZ (blue curve) and TMZ-LfNPs (red curve). No of animals per treatment group = 6.

The tumor volume and tumor area also showed a significant decrease in TMZ-LfNP-treated mice **(**Fig. 7A and B, **grey bar)** as compared to mice treated with free TMZ **(**Fig. 7A and B, **white bar)**. A PI/annexin V-FITC staining of brain tumor **(**Fig. 7C **)** suggested an increased apoptotic cell percentage in TMZ-LfNP treated mice **(**Fig. 7C, **gray bar)**, as compared to mice treated with TMZ **(**Fig. 7C, **white bar)**. The enhanced apoptotic activity was further confirmed by studying caspase-3 activation through western blotting. A higher intensity band of cleaved caspase-3 was observed in mice treated with TMZ-LfNPs, as compared to free TMZ **(**Fig. 7D **)**, suggesting increased apoptotic activity.

In a separate survival curve studies, the glioma bearing mice were randomly divided in separate groups (n = 6) and treated as given in method sections. Their life-spans were recorded and plotted using Kaplan-Meier survival estimates **(**Fig. 7E **)**. The median survival time of PBS treated mice was 10 days **(**Fig. 7E, **black curve)** as compared to 18 days when treated with TMZ **(**Fig. 7E, **blue curve)**. However, the increase in median survival was found to be highest (25 days) in the mice treated with TMZ-LfNPs **(**Fig. 7E, **red curve)**. The increase in survival time (IST) of the group treated with TMZ-LfNPs was found to be 2.5 times more as compared to PBS treated group and 1.4 times more than free TMZ treated group. Our results indicate that the improved anti-tumor activity of TMZ-LfNPs leads to a significant enhancement in median survival time of glioma bearing mice.

### Toxicity studies

For toxicity studies the blood samples were collected at the end of treatment from the glioma mice and various blood parameters were estimated. In free TMZ treated animals a significant reduction in the number of immune cells *viz*. leukocytes, lymphocytes and platelets was observed. Also, an increase in the levels of enzyme markers for cardiotoxicity, hepatotoxicity, nephrotoxicity *viz*. lactate dehydrogenase (LDH), serum glutamic oxaloacetic transaminase (SGOT), serum glutamic pyruvic transaminase (SGPT), creatinine and blood urea nitrogen (BUN) in the TMZ-treated group was observed, revealing the toxic side-effects of the drug. The levels of these blood parameters and serum enzymes in the TMZ-LfNP-treated mice were however found to be similar to the untreated or normal mice. Our results, as summarized in Supplementary Table 2, suggest that TMZ delivery through LfNPs substantially reduces its side effects. Similar studies were reported by our group where drug related toxicity was minimized upon encapsulation in Lf nanoparticles^22^.

## Discussion

The efficient encapsulation of TMZ in nanoparticles and its delivery across the BBB has been a challenge. The ability of Lf to cross BBB and its applications in drug/gene delivery has been previously exploited through functionalization of nanoparticles with Lf^31–34^. However, for the first time, utilization of Lf as a sole matrix for developing nano-formulations was reported by our group^21, 24, 25^. In the present study, TMZ loaded Lf nanoparticles were developed and their brain targeting abilities were evaluated which till now has remained unexplored. NPs, reported in the present study, demonstrate efficient drug loading and brain targeting capabilities. The advantage of using Lf-based nanoformulations is the utilization of minimal number of components and absence of any derivatization steps^25^. Such “as is” formulations are more acceptable in federal regulatory demands. The classic example is nab-paclitaxel (Abraxane®)^35^, a FDA approved nanoformulations of human serum albumin (HSA), wherein paclitaxel bound with protein through hydrophobic interaction without involving any chemical conjugation^35, 36^. It is a formulation of choice for most cancers due to its simple preparation and excellent efficiency.

The LfNPs used in this study have several advantages. Firstly, the preparation of TMZ-LfNPs does not involve any heating^37^ or cross-linking^38^, unlike the other reported methods of stabilization of protein nanoparticles used in drug delivery^39, 40^. Furthermore glutraldehyde, a cross linker used in stabilizing protein nanoparticles, requires additional toxicity evaluation as it is toxic^41^. Compared to other methods of protein nanoparticle preparation, *viz*. coacervation^42^, salt-precipitation, heat denaturation^43^ etc, our method is non-disruptive and maintains the physiological conformation of the Lf protein thereby preserving its receptor binding properties^24^. Also, developed from a natural protein (Lf), LfNPs were found to be completely safe even at high doses. Secondly, a high TMZ entrapment efficiency of ~45%, which is significantly higher from that reported earlier (~5%)^13, 26, 27^, could be achieved probably due to strong hydrophobic interactions between Lf protein and TMZ. Thirdly, the protocol optimised for the preparation of LfNPs is highly reproducible in terms of nanoparticle properties and entrapment efficiencies as reported earlier^21, 23^. Though LfNPs show near neutral surface charge, their stability is probably due to strong hydrophilic nature of the particle surface contributed mainly by both positive and negative fixed charges from side groups of amino acids. Unlike charged nanoformulations, low zeta potential of these nanoparticles may contribute to the enhanced circulation life of the particles by reducing opsonisation through mononuclear phagocytic system (MPS)^44, 45^. Also, the size of LfNPs obtained in the present study (160 nm), is considered to be particularly advantageous for passive accumulation in tumor tissue due to the EPR effect^46^. The observed stability of LfNPs, as assessed by DLS, may also contribute to the longer *in vivo* half-life. Further, the FT-IR spectra suggest the stability of TMZ and Lf protein in the nanoformulations, as the characteristic peaks corresponding to TMZ and Lf are observed in the nanoformulation as well. Fourthly, LfNPs show a surprising ability to release TMZ (4-fold excess) at acidic pH, a phenomenon observed for several other drugs by our group^21, 24^. This property may facilitate a triggered TMZ release within the acidic tumor interstitium and in the endosomal compartments, following LfR-mediated endocytosis of LfNPs. The prolonged retention of intracellular drug when delivered as nanoformulation reported in many studies^23, 47, 48^ is probably due to the reduced efflux kinetics of nanoform of drug as compare to free drug. The Lf receptor blocking studies suggest that the particle uptake process is largely determined by the receptor endocytotic process. The preferential release of TMZ from TMZ-LfNPs at lower pH could be attributed to the pH dependent changes in the secondary structure of lactoferrin^49^. These structural changes may lead to a reduction in nanoparticle size thereby releasing the encapsulated drug. The receptor-mediated uptake of LfNPs contributed to an enhanced intracellular TMZ concentration and prolonged intracellular retention of TMZ, leading to a significant reduction (10-fold) in IC_50_ values of TMZ on GL261cells. This gives the advantage of using reduced dosage of TMZ in the nanoformulations thereby minimising unwanted side effects and resistance due to prolonged dosing^9^.

The brain distribution studies of healthy mice showed an enhanced accumulation of the drug. The concentration of TMZ in brain was found to increase by 3-fold when delivered *via* nanoparticles. Similarly, the plasma bioavailability studies exhibited an improved PK profile - AUC (area under the curve) and T_1/2_ showed an increase by 5-fold and 2-fold respectively, when compared to free TMZ. The significantly higher brain accumulation of TMZ may be attributed to the plasma stability of TMZ-LfNPs and the over-expression of LfR in brain and endothelial tissue which facilitates efficient crossing of BBB by TMZ-LfNPs and accumulation in the brain.

Tumor regression abilities of NPs were studied in intracranial orthotopic glioma mice model. As compared to subcutaneous xenograft, intracranial orthotopic tumor models are considered to be more relevant in order to obtain accurate response to therapies^50, 51^. Moreover, GL261based glioma model is considered more appropriate for studying human gliomas due to its infiltrative nature and also for carrying key mutations in p53 and K-ras genes that are similar to human gliomas^52^. Brain distribution studies in glioma mice model showed a preferential and more than 3-fold accumulation of TMZ at the tumor site when delivered through LfNPs; as compared to free TMZ. This could be attributed to the combined effects of passive accumulation, due to enhanced permeability and retention effect (EPR effect), and active targeting due to preferential binding with over-expressed LfRs on the glioma tissue. The higher drug concentration at the tumor site ultimately led to significantly higher anti-tumor activity in TMZ-LfNPs treated group as assessed by reduced tumor volume and significantly higher tumor cell apoptosis. The overall pharmacological benefit of TMZ in TMZ-LfNPs showed a significant improvement in survival index of glioma bearing mice as compared to treatment with free TMZ. The improved survival of glioma mice either by encapsulating TMZ in tumor targeting nanocomplex^11^ or by sensitizing TMZ chemotherapy has been reported earlier^53, 54^. Additionally in this study, along with previous studies from our group^21^, it has been shown that LfNPs have excellent biocompatible properties and the off-target effects of TMZ are significantly reduced when delivered via LfNPs.

Therapeutic dosage of TMZ is currently limited by the risk of developing myelosuppressive disorders^55^. When delivered *via* LfNPs, TMZ did not result in any alteration in haematological parameters that are otherwise observed with free TMZ. Thus, a clear advantage of LfNP is its potential to deliver effective dosage of TMZ at the glioma site with minimal toxicity.

## Conclusion

The results presented here suggest that exploiting lactoferrin nanoparticles (LfNPs) for their ability to cross BBB, tumor-targeting and pH dependent drug release, provides a means to enhance the therapeutic efficacy of TMZ with reduced side-effects. LfR specific uptake and subsequent toxicity on GL261cells leading to a significant reduction in tumor burden in orthotopic glioma model, substantiating the utility of this formulation in glioma treatment. Prepared from a natural and abundant protein with no modifications, LfNPs were shown to be safe carrier for TMZ. We strongly believe that the ability of the nanoformulation reported here to target tumors with a high degree of specificity may allow dose escalation and result in an improved response in glioma patients along with increasing the median life survival. Moreover, the proof-of-concept study provided here may be applied for other drugs.

## Methods

### Preparation of TMZ-loaded Lf-nanoparticles

Temozolomide-loaded Lf-nanoparticles (TMZ-LfNPs) were prepared by a previously described sol-oil method with minor modifications^22, 24^. Briefly, TMZ dissolved in DMSO (~25 mg/ml) was mixed with Lf prepared in phosphate-buffered saline (PBS) at 1:2 (w/w) ratio and incubated for 30 min on ice, followed by addition of 20 ml olive oil. The mixture was sonicated with a 30 s pulse followed by cooling for 1 min (Power range 50–80%). The cycle was repeated 15 times with temperature maintained at 4 °C. The sonicated mixture was snap frozen in liquid nitrogen and then thawed on ice. The suspension was centrifuged at 30,000 × g for 15 min and the pellet, containing the particles, was washed thrice with diethyl ether followed by three washes with phosphate-buffered saline (PBS) to remove excess oil and free TMZ. Finally, the particles were dispersed in PBS and stored at 4 °C till further use. Alternately, the particles were dispersed in double distilled water, snap frozen and lyophilized. Fluorescein (FL)-loaded Lf-nanoparticles (FL-LfNPs) were prepared using the above mentioned method except that FL was used in place of TMZ.

### Characterization of Nanoparticles

For TEM analysis, LfNPs were resuspended in 0.01 M PBS (pH 5.5 or 7.4). The particles were then placed on carbon coated copper 300 mesh grids, air dried and stained using 1% aqueous solution of uranyl acetate for 1 min. The samples were examined using Transmission Electron Microscope (JEM-2100, M/S Jeol Limited, Tachikawa, Tokyo, Japan).

The hydrodynamic diameter, polydispersity index (PDI) and zeta potential were analysed by SZ-100 Nanopartica analyzer system (Horiba Scientific, USA) equipped with a Diode-pumped solid-state (DPSS) laser of wavelength 532 nm. Measurements were made by suspending the particles in 0.01 M PBS (pH 5.5 or 7.4).

For, *in vitro* stability studies, TMZ-LfNPs were prepared as described earlier, and stored in PBS at 4 °C. At regular time intervals, aliquots were withdrawn, mildly sonicated and analyzed for their hydrodynamic diameter using DLS as described above.

### Determination of drug encapsulation efficiency and drug loading content

TMZ entrapment efficiency in nanoparticles was determined by resuspending 0.5 mg TMZ-LfNPs in 1 ml of PBS (pH 5.0) and incubated for 4 h at 37 °C on a rocker. 100 µl of 20% AgNO3 was then added to precipitate the lactoferrin protein, followed by addition of 900 µl of mobile phase (a mixture of water: methanol: acetonitrile (45:45:10 v/v), pH adjusted to 3.0 with o-phophosphoric acid). The mixture was centrifuged at 30,000 × g for 15 min, supernatant was collected and filtered through a 0.2 µm syringe filter. The amount of TMZ released was quantified using an Agilent 1200 series reversed-phase high performance liquid chromatography (RP-HPLC) system. Separation was carried out by using a Zorbax 300 SB-C18 column (250 mm × 4.6 mm, 5 µm particle size, Flow rate - 0.5 ml min^−1^, Injection volume - 200 µl and run time - 10 min). TMZ was estimated by its absorption at 340 nm. The limit of detection of TMZ by this method was found to be 10 ng ml^−1^. All experiments were performed in triplicates. TMZ entrapment efficiency and drug loading content was calculated by the following formulae.

1 $${\rm{Encapsulation}}\,{\rm{efficiency}}\,( \% )=\frac{({\rm{Mass}}\,{\rm{of}}\,{\rm{the}}\,{\rm{drug}}\,{\rm{in}}\,{\rm{NP}})}{(\text{Initial}\,{\rm{mass}}\,{\rm{of}}\,{\rm{drug}}\,\text{used})}\times 100$$

2 $${\rm{Drug}}\,{\rm{loading}}\,{\rm{capacity}}=\frac{({\rm{Mass}}\,{\rm{of}}\,{\rm{the}}\,{\rm{drug}}\,{\rm{in}}\,{\rm{NP}})}{({\rm{Total}}\,{\rm{mass}}\,{\rm{of}}\,{\rm{the}}\,{\rm{NP}})}\,\times 100$$

### *In vitro* TMZ release studies

NPs (0.5 mg) were resuspended in different pH values (1–8) of 1 ml of 0.01 M PBS and incubated for 4 h at 37 °C on a rocker. 100 µl of 20% AgNO3 was then added and followed by addition of 900 µl of mobile phase. Samples were collected by centrifugation at 30,000 × g for 15 min. TMZ released from the nanoparticles was quantified using HPLC as described in supplementary information.

To study the release kinetics of TMZ from TMZ-LfNPs, the nanoparticles (10 mg ml^−1^) were resuspended in PBS (pH 5.5 or 7.4) and placed in dialysis tubings (MWCO 8 kDa to 12 kDa) and incubated in an incubator shaker at 37 °C. Samples were collected at various time intervals (0 to 72 h) and analyzed for TMZ using HPLC as described in supplementary information.

### Cell culture

All the cells (GL261, HepG2 and A549) were maintained in a growth medium composed of Dulbecco’s modified Eagle’s medium (DMEM, Invitrogen) supplemented with 10% fetal bovine serum (Invitrogen), 5 μg ml^−1^ penicillin, 6 μg ml^−1^ streptomycin and 10 μg ml^−1^ kanamycin. Cells were grown and maintained in 5% CO_2_, 95% relative humidity at 37 °C.

### Receptor-mediated endocytosis of TMZ-LfNPs

Lf receptor (LfR) expression in GL261 cells was determined by western blot. Briefly, GL261 cells along with positive (HepG2) and negative control (A549) cells were homogenised in radio-immuno-precipitation assay buffer (RIPA: 50 mM Tris-Cl pH 8.0, 150 mM NaCl, 2 mM EDTA, 1% (w/v) NP-40, 0.5% (w/v) sodium deoxycholate, 0.1% (w/v) SDS containing protease inhibitor cocktail (G-Biosciences, India). The homogenate was subsequently centrifuged to remove insoluble debris and protein content of the lysate was estimated using Bradford’s method. 20 μg of protein was then resolved using 12% SDS-PAGE and transferred to a nitrocellulose membrane. The membrane was subsequently blocked with 5% BSA and probed with primary anti-LfR antibody followed by secondary antibody conjugated with horseradish peroxidase. The blot was developed by a Vilber-Lourmat Chemiluminescence Imaging System using the Chemi-Capt software, after adding the Pierce^®^ ECL Western Blotting HRP substrate (Thermo Scientific). To investigate the role of LfR in the cellular internalization of LfNPs, a receptor blocking experiment was performed. The uptake of LfNPs was assessed by pre-incubating cells with anti-LfR antibodies (1:300) in serum-free medium for 1 h. Subsequently FL-LfNPs (100 µg ml^−1^) were added to the cells and further incubated for 2 h. After incubation, the cells were processed for confocal microscopy as mentioned previously. The effect of receptor blocking on TMZ delivery through LfNPs was investigated using HPLC. Briefly, GL261 cells (1 × 10^6^) were incubated with anti-LfR antibody in serum-free media for 1 h followed by 2 h incubation with TMZ-LfNPs (100 µg ml^−1^). After incubation, cells were washed thrice with PBS and sonicated in PBST for 30 s. Protein in the cell lysate was precipitated by adding equal volumes of 20% silver nitrate (AgNO_3_) and incubated overnight at 4 °C. The precipitated protein was separated by centrifugation at 11000 × g for 15 min at 4 °C and TMZ was quantified using HPLC as mentioned previously.

### Animal experiments

All animal experiments were conducted in accordance with protocols approved by the Institute’s Animal Ethics Committee of Centre for Cellular and Molecular Biology, which was constituted by the national body, Committee for the Purpose of Control and Supervision of experiments on Animals, (Project numbers 41/2016). Adult C57BL/6 mice (22–24 g) were maintained at 22 °C on a 12 h light and dark cycle in polyethylene cages with stainless steel lids with *ad libitum* access to food and water. Glioma generation was performed under anaesthesia using intraperitonial (i.p) injection of a mixture of Ketamine (120 mg kg^−1^) and xylazine (6 mg kg^−1^) in saline. For survival studies, 6 animals in each group and for all other *in vivo* experiment, 3 animals each group were utilized.

### BBB crossing capability and brain localization studies of LfNPs in healthy mice

9 animals were randomly divided in three groups (n = 3) and intravenously injected with (i) PBS, (ii)100 µl of free Lf (2 mg ml^−1^) and (iii) LfNPs (equivalent dose of free Lf). After 24 h, hearts of the treated mice were perfused with 4% paraformaldehyde. Subsequently, brain tissues were removed, snap frozen in liquid nitrogen and stored at −80 °C till further use.

For immunohistochemistry, frozen brain tissues were embedded in OCT tissue freezing medium (Leica Biosystems) and cryosectioned to obtain 5 µm thick sections on positively charged slides. The tissue sections were fixed in isopropanol for 5 min and incubated with anti-Lf antibody at room temperature (RT) for 1 h, followed by staining with Alexa Fluor-488 labelled secondary antibody. The sections were counterstained with propidium iodide (PI) and visualized under a confocal fluorescence microscope.

For transcytosis studies, mice were intravenously injected with 100 µl of FL-LfNPs (2 mg ml^−1^). Brain sections, obtained by the procedure described earlier, were blocked in 5% BSA for 1 h followed by incubation with CD31 antibody diluted in PBS containing 0.05% Tween 20 (1:300) for 12 h at 4 °C. The sections were washed and incubated with a Cy3-conjugated secondary antibody and processed as described above. Autofluorescence was removed by treating brain sections with freshly prepared 1% (w/v) NaBH_4_ in PBS for 20 min before immunostaining. Subsequently, the sections were incubated in 1 mM cupric sulphate and 50 mM ammonium acetate solution (pH 5.0) for 1 h^56^.

### Pharmacokinetic studies

C57BL/6 mice were intravenously administered with free TMZ and TMZ-LfNPs (with an equivalent TMZ dose of 10 mg kg^−1^ body weight), *via* tail vein injection. 200 µl of blood was collected by retro-orbital plexus at indicated time points, 0.083, 0.25, 0.5,1, 2, 4, 8 and 24 h following injection. Plasma was separated from the blood samples, followed by 30 min incubation with equal volume of 20% AgNO_3_ at room temperature (RT). Subsequently, the homogenates were centrifuged at 11000 × g for 15 min at 4 °C and the supernatant was collected and filtered using a 0.2 μm syringe filter and assayed for TMZ concentration using HPLC as mentioned earlier. At 24 h mice were euthanized and brain was collected and assessed for TMZ uptake using HPLC. Pharmacokinetic parameters from quantified TMZ were calculated using Kinetica V.5.0 software with non-compartmental model of estimation.

### Orthotopic glioma model generation

Tumor was developed by intracranial implantation of GL261 cells ^57, 58^. Briefly, C57BL/6 mice (21–22 gm) were anesthetised by i.p. injection of Xylene/Ketamine and cells (1 × 10^4^ cells in 3 μl PBS) were injected stereo-tactically into the right striatum (1.8 mm lateral to the bregma at 3 mm depth) using a Hamilton syringe (Harvard Apparatus, UK) along with a 26-gauge needle at 0.2 μl min^−1^ attached to a stereotaxic frame (Stoelting, USA). Wounds were closed with sutures and mice were carefully monitored until recovery from anaesthesia.

### *In vivo* tumor targeting studies

The ability of systemically administered LfNPs to deliver TMZ at the tumor site was evaluated using HPLC. Six Glioma bearing C57BL/6 mice were randomly divided in two groups (n = 3) and treated with free TMZ (5 mg kg^−1^ body weight) and equivalent doses of TMZ in TMZ-LfNPs. Different parts of the brain tissue were isolated and TMZ was quantified using HPLC as described above.

### *In vivo* tumor regression and survival studies of TMZ-LfNPs in glioma bearing mice

Nine intracranial glioma bearing mice, were randomly divided into three groups (n = 3) and injected with: (i) PBS (ii) TMZ (5 mg kg^−1^ body weight) and (iii) TMZ-LfNPs (equivalent dosage of TMZ) on 3^rd^, 5^th^, 7^th^ and 9^th^ day as described in the schedule given (Fig. 6A). When all the PBS treated mice succumbed to death (14^th^ day after the GL261 cells implantation), brain tissues from all the experimental groups were collected and fixed with 10% formalin (48 h). The tissues were then embedded in paraffin wax followed by sectioning (5 µm sections) and staining with Haematoxylin & Eosin (H & E) using standard protocols. The tumor area in the histology sections was measured using Image J software. To calculate the tumor volume (V), the maximum diameter (a) and minimum diameter (b) of tumor mass in the whole brain tissue was measured using vernier caliper and the tumor volume was calculated using the below formula.

$${\rm{Tumor}}\,{\rm{volume}}\,({\rm{V}})=0.5\,{{\rm{ab}}}^{2}$$

For survival curve analysis, another set of 18 intracranial glioma bearing mice were randomly divided into three groups (n = 6) and treated as above in accordance with the treatment schedule (Fig. 6A) and monitored for their survival till they became moribund. Survival data was analyzed with log-rank test in Kaplan Meier non-parametric analysis mode.

The *in vivo* response to TMZ-LfNPs was assessed by quantifying the level of apoptosis in tumor tissue using FITC Annexin V/dead cell apoptosis kit (Molecular probes). Briefly, the glioma bearing mice, after treatment with either PBS, TMZ or TMZ-LfNPs as described earlier were euthanized and the tumor bearing brain was dissected. Single-cell suspensions were obtained by collagenase digestion^59^. Subsequently cells were passed through a 70 μm cell strainer (BD Falcon), stained according to the supplier’s protocol and analyzed using flow cytometry (Gallios, BecKman Coulter). Gating on forward and side scatter was done to exclude cell debris and 10,000 gated events were recorded. Data was analysed using Kaluza flow cytometry analysing software provided by the supplier.

Apoptosis was further analyzed by determining the levels of cleaved Caspase-3 using western blot. Briefly brain tumor lysate was processed as mentioned earlier and 20 μg of protein was resolved on a 12% SDS-polyacrylamide gel, transferred to nylon membrane and probed with antibody against Caspase-3 followed by secondary antibody conjugated with horseradish peroxidase and the blot was developed as described above.

### Toxicity studies in glioma bearing mice

Toxicity related parameters were tested at the end of the treatment in glioma bearing mice (schedule and dosages are as described above) by collecting blood samples for haematological and serum chemistry analysis. The complete blood picture from untreated control and treated mice was obtained using haematology analyzer Elite 5, as per manufacturer’s protocol. The following enzymes were tested for hepatotoxicity; alanine aminotransferase (ALT), aspartate aminotransferase (AST) and alkaline phosphatase (ALP). For nephrotoxicity creatinine (CRE) and blood urea nitrogen (BUN) were studied. Lactose dehydrogenase (LDH) was monitored for cardiotoxicity. Activity of all above mentioned enzymes were measured by using biochemical kits as per the manufacturer’s protocol (Tulip Pvt. Ltd.).

### Statistics

The statistical significance was determined by using two-tailed unpaired student’s t test. P-values of <0.05 were considered significant. Statistical significance for Kaplan Meier curves was determined by log rank analysis. All values were expressed as the Mean ± Standard Deviation (S.D) of triplicate experiments. Data analysis was performed in Microsoft Excel and Origin Pro 8 software from Origin Lab.

## Electronic supplementary material

Supplementary information
