# Supplementary material for: Overcoming blood brain barrier with a dual purpose Temozolomide loaded Lactoferrin nanoparticles for combating glioma (SERP-17-12433)
Source: Sci Rep. 2017 Jul 26;7:6602. doi: 10.1038/s41598-017-06888-4 (PMC5529576; doi:10.1038/s41598-017-06888-4)
Supplement: Supplementary file 1 — Supplementary information [file 41598_2017_6888_MOESM1_ESM.doc]

# SUPPLEMENTARY INFORMATION

**(SERP-17-12433)**

**Overcoming blood brain barrier with a dual purpose Temozolomide loaded Lactoferrin nanoparticles for combating glioma**

Sonali Kumari^a^, Saad M. Ahsan^b^, Jerald M. Kumar^b^, Anand K. Kondapi^a*^ and Nalam M. Rao^b*^

*^a^ Department of Biotechnology and Bioinformatics, School of Life Sciences, University of Hyderabad, Prof. C. R. Rao Road, Gachibowli, Hyderabad 500 046, Telangana State, India*

*^b^Centre for Cellular and Molecular Biology (CCMB), Council of Scientific and Industrial Research, Uppal Road, Hyderabad 500 007, Telangana State, India*

*Corresponding authors

Email: [madhu@ccmb.res.in](mailto:madhu@ccmb.res.in), akondapi@gmail.com

Address: Dr. Nalam M. Rao

Centre for Cellular and Molecular Biology (CCMB),

Hyderabad 500007, India

Ph.No.- +91-40-27192504

Dr. Anand K. Kondapi

Department of Biotechnology and Bioinformatics,

School of Life Sciences,

University of Hyderabad

Prof. C. R. Rao Road,

Gachibowli, Hyderabad 500 046,

Telangana State, India

Ph.No.- +91-40-23134731

## **Materials**

Lf and olive oil were purchased from Symbiotic, USA and Leonardo, Italy respectively. Temozolomide (TMZ) was a gift from Celon Laboratories Pvt. Ltd (India). Fluorescein (FL) was procured from Sigma (St. Louis, MO). 3-(4, 5-dimethylthiazol-2-yl)-2, 5-diphenyl tetrazolium bromide (MTT) was purchased from Calbiochem (USA). Mouse glioma (GL261), human liver cancer (HepG2) and lung carcinoma (A549) cell lines were obtained from ATCC. Vectashield Mounting Medium with DAPI was procured from Vector Laboratories Inc. (USA). Anti-Lactoferrin (Anti-Lf) and anti-Lf receptor (Anti-LfR) antibodies were purchased from Pierce, Thermo Scientific (USA) and Biorbyt (UK), respectively. Anti-Caspase-III, anti-CD31 and goat anti-rabbit IgG antibodies were purchased from Abcam (UK). FITC Annexin V/dead cell apoptosis kit was purchased from Molecular Probes, Invitrogen (USA). Kits for biochemical analysis were purchased from Tulip Pvt. Ltd. (India). All reagents used in the study were of analytical grade or more.

## Fourier Transform Infra-Red Spectroscopy (FTIR) analysis

The infrared spectra of free Lf, LfNPs, free TMZ and TMZ-LfNPs were obtained at a resolution of 4 cm^-1^ in a FTIR spectrometer (Bruker Alpha T, Bruker Optik, Ettingen Germany), equipped with a single reflection diamond attenuated total reflectance accessory. A drop of the sample was air dried on the surface of diamond crystal element followed with recording of 64 scans at 200 cm^-1^/ min and averaged.

## Estimation of intracellular TMZ

The cellular uptake of TMZ was determined by treating cells with free TMZ and TMZ-LfNPs (equivalent to 60 µg TMZ) for a period of 2 h. The cells were washed with PBS and further incubated in fresh culture media. At indicated time points (0-24 h), cells were harvested. TMZ was estimated from fixed number of cells as described earlier.

## Cytotoxicity assay

The cellular toxicity of TMZ-LfNPs in GL261 cells was estimated by MTT based cell death assay. Briefly, GL261 cells were seeded in a 96-well plate at a density of 5000 cells per well and after obtaining the desired 40-50 % confluency, cells were incubated with increasing concentrations of either free TMZ or TMZ-LfNPs with an equivalent dose of (0 - 200 µg ml^-1^) of TMZ and further incubated for 24 h. Cell viability was calculated using the following formula:

Viability (%) = (A_570nm_ of treated cells / A_570nm_ of control cells) X 100

Inhibition (%) = ((A_570nm_ of control cells - A_570nm_ of treated cells) / (A_570nm_ of control cells)) X 100

**Table 1:** Major FT-IR peaks observed for Lf, LfNPs, TMZ and TMZ-LfNPs.

| **Functional Groups** | **Lf** | **LfNPs** | **TMZ** | **TMZ-LfNPs** |
| --- | --- | --- | --- | --- |
| **Amide I (C=O)** | 1636 | 1662 | ----- | ----- |
| **Amide II (C-N and N-H bending)** | 1539 | 1541 | ----- | ----- |
| **C-O-C** | 1069 | 1072 | ----- | ----- |
| **C=C** | ----- | ----- | 1673 | 1662 |
| **C=N** | ----- | ----- | 1739 | 1745 |

All values reported in unit cm^-1^.

**Table 2:** Effect of TMZ and TMZ-LfNPs on haematological and biochemical parameters in glioma bearing mice

|  | **Untreated** | **TMZ** | **TMZ-LfNPs** |
| --- | --- | --- | --- |
| **Blood Count** | | | |
| **Leukocytes (x10^9^/L)** | 8.27 ± 0.86 | 6.23 ± 0.36 **(L)** | 8.98 ± 0.62 |
| **Lymphocyte (%)** | 47.5 ± 1.25 | 40 ± 0.45 **(L)** | 68 ± 3.60 |
| **Thrombocytes (x10^9^/L)** | 982.00 ± 41.00 | 600 ± 25.00 **(L)** | 1044.67 ± 32.13 |
| **Blood Chemistry** | | | |
| **ALP (U/L)** | 100 ± 6.42 | 70.40 ± 9.34 **(L)** | 93.80 ± 13.46 |
| **ALT (U/L)** | 39 ± 4.36 | 63.20 ± 3.62 **(H)** | 33.40 ± 1.86 |
| **AST (U/L)** | 197 ± 46.50 | 300 ± 46.07 **(H)** | 98.20 ± 25.85 |
| **BUN (mg dL^-1^)** | 17 ± 2.00 | 21.20 ± 0.73 **(H)** | 17.60 ± 1.03 |
| **Creatinine (mg dL^-1^)** | 0.15 ± 0.05 | 0.13 ± 0.10 | 0.12 ± 0.02 |
| **LDH (U/L)** | 75 ± 0.45 | 180 ± 0.23 **(H)** | 82 ± 2.33 |

ALP: alkaline phosphatase, ALT: alanine transaminase, AST: aspartate aminotransferase, BUN: blood urea nitrogen, LDH: lactose dehydrogenase. L (lower) and H (higher) represent more than 25% change from the untreated base line. Values are reported as mean ± S.D for 3 mice in each group.

**Supp-Fig 1**

**
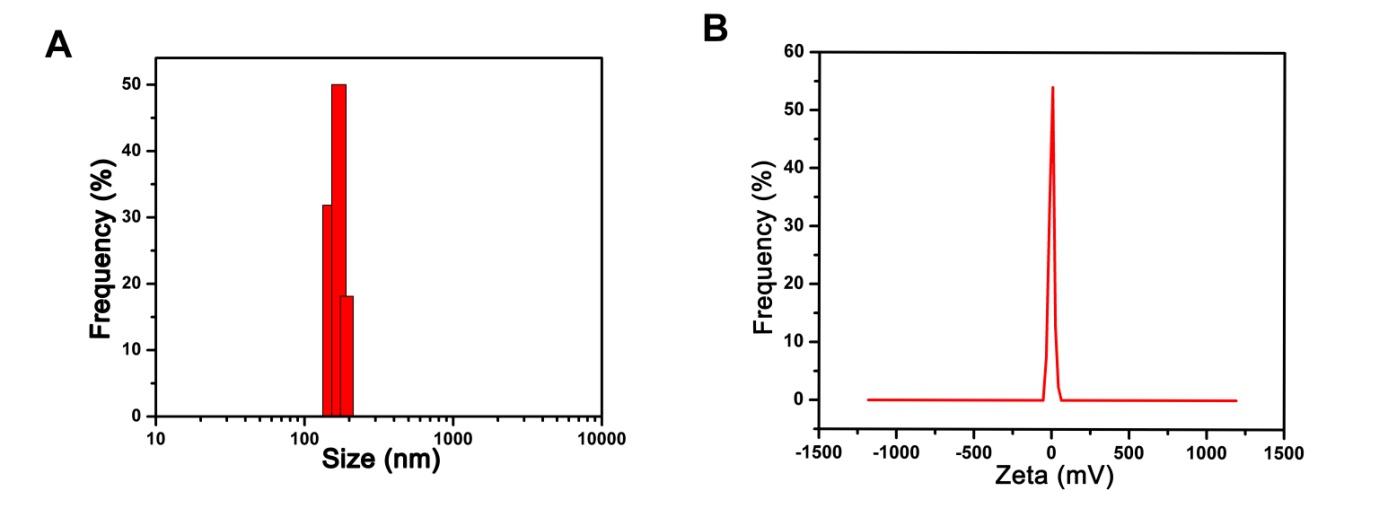
**

**Supp-Fig 1:** A) Hydrodynamic diameter and (B) Zeta potential of FL-LfNPs at pH 7.4

**Supp-Fig 2**

**Supp-Fig 2:** Stability of TMZ-LfNPs. Black bars indicate the size (hydrodynamic diameter) of the particles stored in PBS at 4 °C and the red bars represent the size of particles stored in the presence of 10% serum and at 4 °C. Each data point was replicated three times and presented as Mean ± Standard Deviation (S.D).

**Supp-Fig 3**


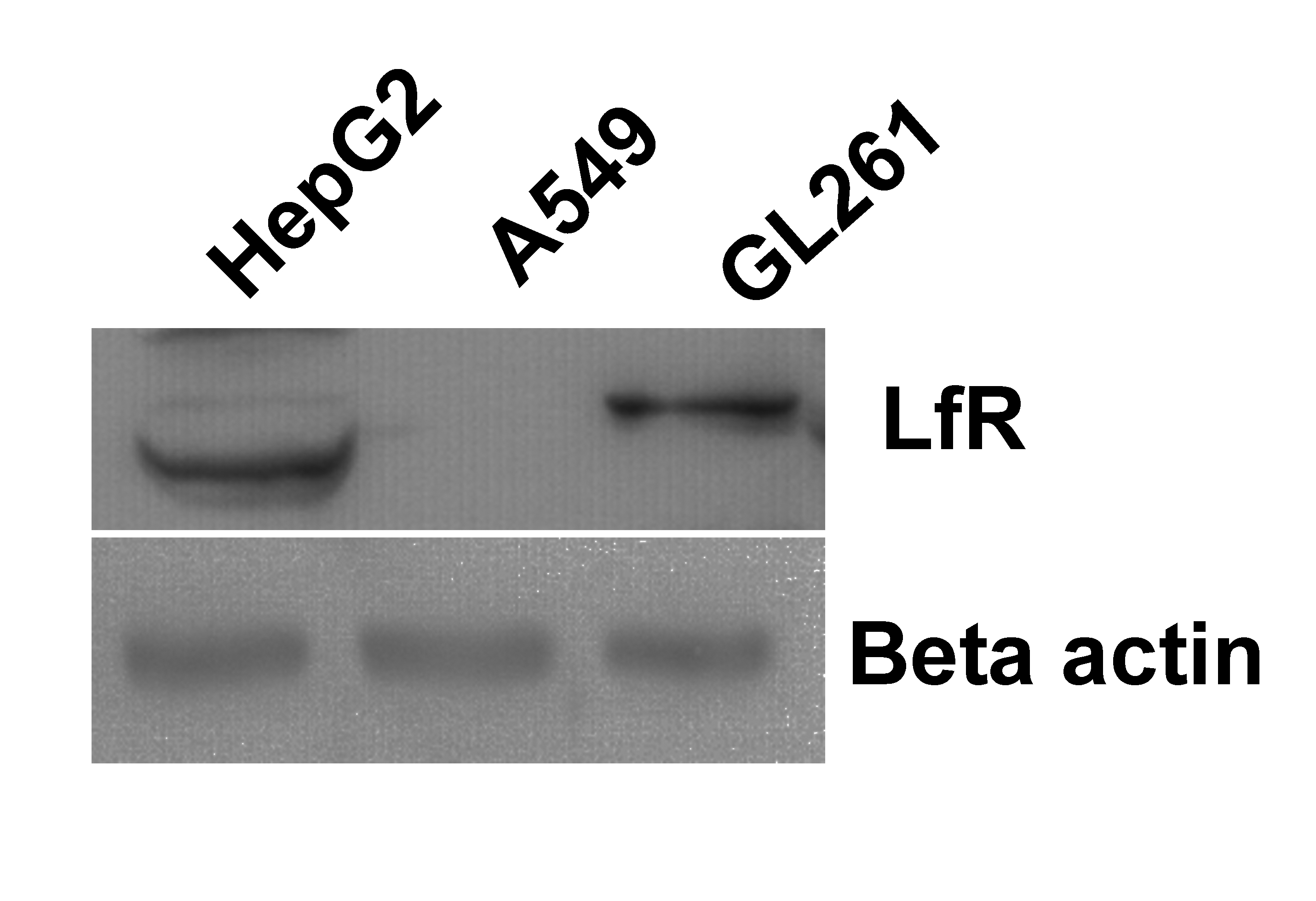


**Supp-Fig 3:** Western blot for LfR expression in HepG2, A549 and GL261 cells.

**Full length blots of Figure 7 Caspas-3**

**
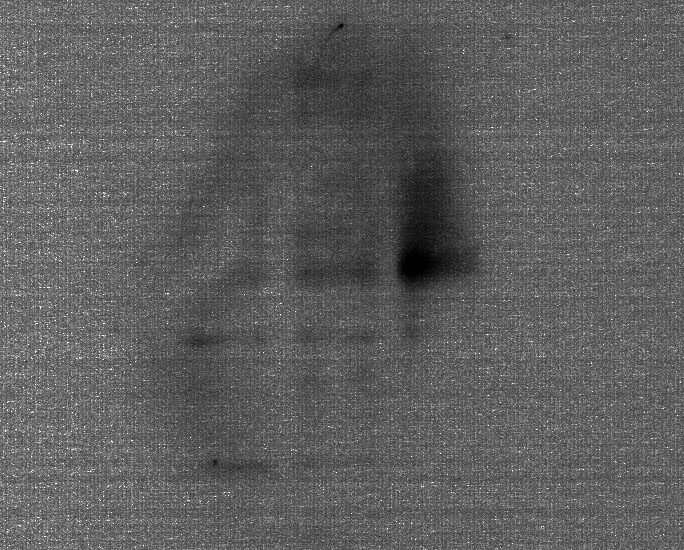
**

**Full length blot of Figure 7 GAPDH**

**
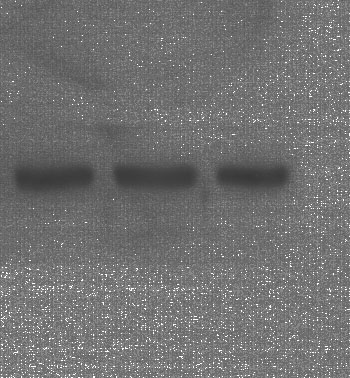
**
